# Supplementary figures and images for: Visualizing molecules of functional human profilin
Source: eLife. 2022 Jun 6;11:e76485. doi: 10.7554/eLife.76485 (PMC9249392; doi:10.7554/eLife.76485)

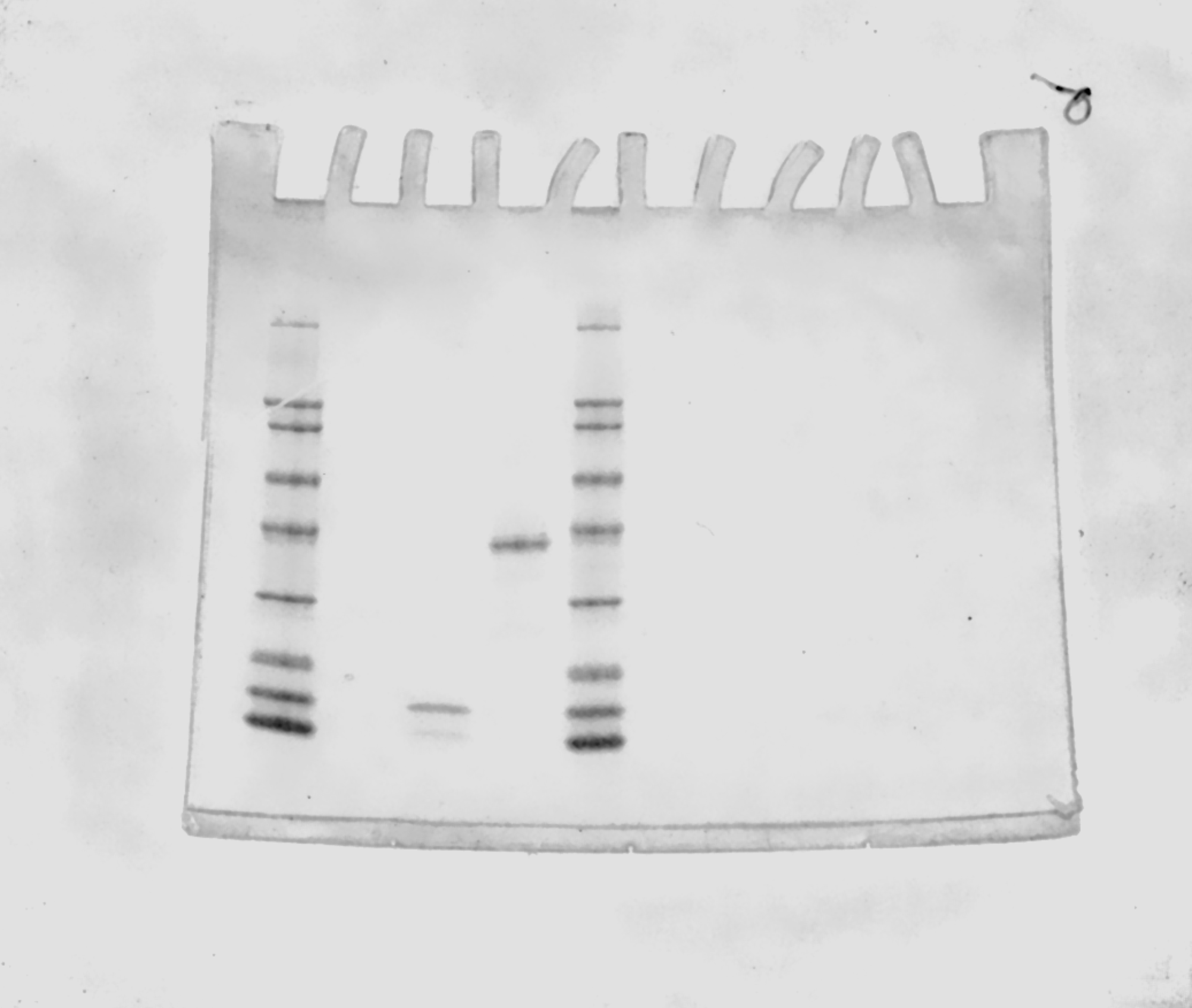

Supplement: Figure 1—source data 1. [file elife-76485-fig1-data1.zip › Figure 1/Figure 1 source file.tiff]

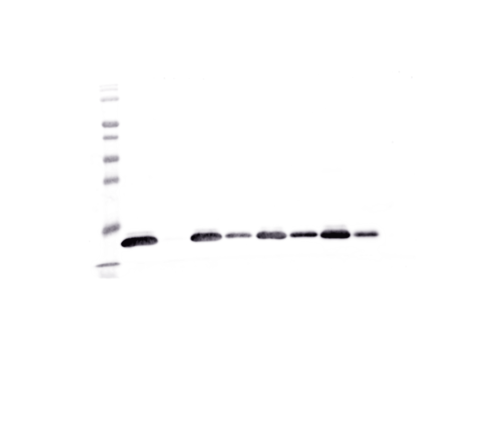

Supplement: Figure 2—source data 1. [file elife-76485-fig2-data1.zip › Figure 2 and F2SF1_source data/Figure2B_source_data.tiff]
